# Interpretations about gender in the epidemiology of cutaneous melanoma^☆^

**DOI:** 10.1016/j.abd.2022.01.001

**Published:** 2022-03-21

**Authors:** Anna Carolina Miola, Juliano Vilaverde Schmitt, Hélio Amante Miot

**Affiliations:** Department of Dermatology and Radiotherapy, Faculdade de Medicina, Universidade Estadual Paulista, Botucatu, SP, Brazil

Dear Editor,

Due to the relevance of cutaneous melanoma to the health system and the scarcity of large series of cases with longitudinal follow-up in samples from the Brazilian population, we read with great interest the article by Castro e Souza et al.,^1^ which showed different survival rates in males and females, in a five-year follow-up of 221 patients. In addition to congratulating the authors, we would like to propose some reflections regarding the interpretation of the results.

There is consistent evidence of an increase in melanoma diagnoses worldwide in recent decades; in parallel, there is an intense discussion about factors associated with this phenomenon, such as population aging, increase in sun exposure during leisure time, use of immunosuppressants, greater population awareness, greater access to the health system, and even greater diagnostic accuracy by dermatologists and pathologists. Nevertheless, the population-specific mortality did not show any signs of decrease, further alluding to the possibility of overdiagnosis.^2^, ^3^

Shorter survival in men with melanoma have also been identified in other countries; however, the tumor biological behavior considering the hormonal differences, as defended by the authors, may not fully justify this fact, especially since the greatest divergence of mortality between the sexes occurs in old age, when sex hormones are less active.^2^, ^4^

It is considered relevant to mention that all diseases have biological, environmental and sociocultural dimensions. In Brazil, women have a life expectancy that is 9.6% higher than men (2019: 80.1 *vs*. 73.1 years), undergo fewer hospital admissions (except for obstetric ones), and have a greater perception of their own health care, such as adherence to vaccination, regular examinations and routine outpatient consultations. In the meantime, behavioral aspects related to masculinity should be considered as possible determinants of delay in the diagnosis of melanoma, contributing to its worse prognosis.

To corroborate this hypothesis, the data from Castro e Souza et al. was reanalyzed regarding the Clark and Breslow levels – considering such variables as ordinals. When analyzing them using the Chi-Square test of tendency, which considers the influence of the effect by the variable order,^5^ it was shown that women have better histopathological indices (p < 0.017) at the moment of diagnosis of invasive cases (Fig. 1).

**Figure 1:**
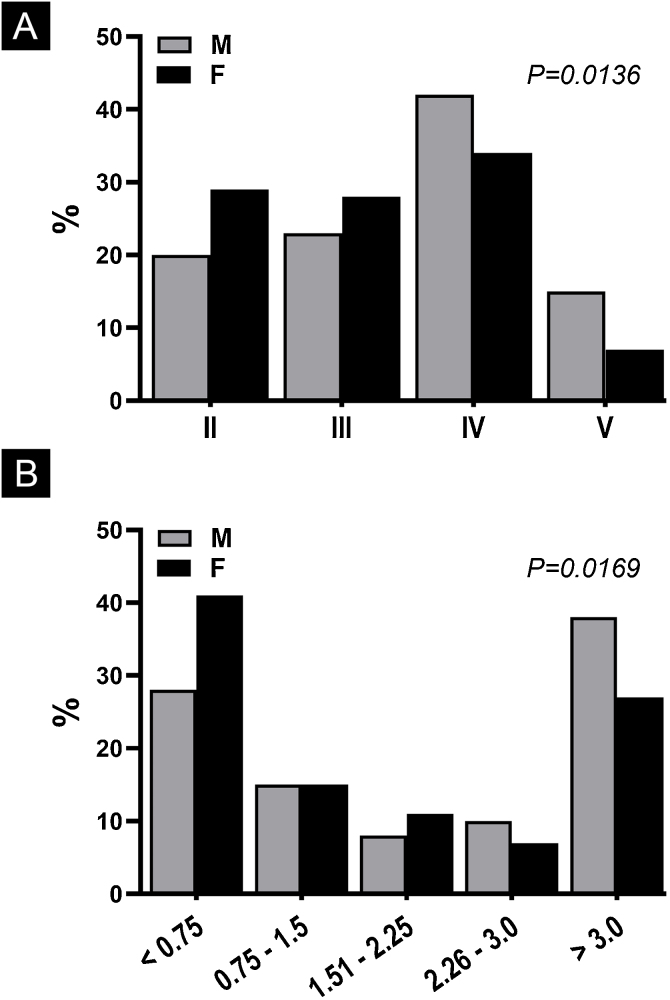
Percentage of cutaneous melanoma cases according to gender (M, Male; F, Female) and level of histopathological invasion. (A), Clark’s anatomical level (n = 267); (B), Breslow index in mm (n = 265). Data extracted from Castro e Souza et al.^1^

In conclusion, the results of Castro e Souza et al. should be seen from the perspective of primary and secondary prevention, aiming to substantiate policies that promote early diagnosis, especially in groups at higher risk and mortality, such as men and the elderly, since the worse melanoma survival in men may also reflect the socio-cultural determinant associated to gender, leading to late diagnoses in groups less aware of the importance of the disease.

## Financial support

None declared.

## Authors’ contributions

Anna Carolina Miola: Design and planning of the study; statistical analysis; drafting and editing of the manuscript; critical review of the literature; critical review of the manuscript; approval of the final version of the manuscript.

Juliano Vilaverde Schmitt: Design and planning of the study; statistical analysis; drafting and editing of the manuscript; critical review of the literature; critical review of the manuscript; approval of the final version of the manuscript.

Hélio Amante Miot: Design and planning of the study; statistical analysis; drafting and editing of the manuscript; critical review of the literature; critical review of the manuscript; approval of the final version of the manuscript.

## Conflicts of interest

None declared.
